# The New and Computationally Efficient MIL-SOM Algorithm: Potential Benefits for Visualization and Analysis of a Large-Scale High-Dimensional Clinically Acquired Geographic Data

**DOI:** 10.1155/2012/683265

**Published:** 2012-03-19

**Authors:** Tonny J. Oyana, Luke E. K. Achenie, Joon Heo

**Affiliations:** ^1^Advanced Geospatial Analysis Laboratory, GIS Research Laboratory for Geographic Medicine, Department of Geography and Environmental Resources, Southern Illinois University, 1000 Faner Drive, MC 4514, Carbondale, IL 62901, USA; ^2^Engineering Building A462, School of Civil and Environmental Engineering, Yonsei University, 262 Seongsanno, Seodaemun-gu, Seoul 120-749, Republic of Korea; ^3^Department of Chemical Engineering, Virginia Polytechnic Institute and State University, Blacksburg 24061, USA

## Abstract

The objective of this paper is to introduce an efficient algorithm, namely, the mathematically improved learning-self organizing map (MIL-SOM) algorithm, which speeds up the self-organizing map (SOM) training process. In the proposed MIL-SOM algorithm, the weights of Kohonen's SOM are based on the proportional-integral-derivative (PID) controller. Thus, in a typical SOM learning setting, this improvement translates to faster convergence. The basic idea is primarily motivated by the urgent need to develop algorithms with the competence to converge faster and more efficiently than conventional techniques. The MIL-SOM algorithm is tested on four training geographic datasets representing biomedical and disease informatics application domains. Experimental results show that the MIL-SOM algorithm provides a competitive, better updating procedure and performance, good robustness, and it runs faster than Kohonen's SOM.

## 1. Introduction

Algorithm development to support geocomputational work has become a key research topic and increasingly has gained prominence among the geocomputational community. This focus area was first inspired by the ground-breaking works proposed by Openshaw et al. [1] and his successive works that emphasized the role of algorithms in geography [2, 3]. Such algorithms include ones for indexing, search, storage, retrieval, display, visualization, and analysis. However, the proliferation of these algorithms and their associated domain-specific applications call for the need to urgently present and develop efficient and effective data clustering as well as visualization tools so as to manage and understand massive digital datasets that are currently being generated through numerous data collection mechanisms. This study sets out to consider a well-known Kohonen's self-organizing map (standard SOM) with a view to improve it in order to make sense of health outcomes associated with the environment. SOM was chosen due in part to its topological ordering and low-dimensional layout and is well documented in SOM clustering literature.

The design and implementation of SOM algorithms to facilitate GIS applications has received considerable attention, especially among the geocomputational community with a keen interest to understand multivariate data. Notable developments started with the conceptualization of standard SOM [4, 5] followed by the development of a variety of applications such as SAM-SOM [6]; interactive and visual exploratory tools [7], Spatialization methods [8], classification of remotely sensed images [9, 10], GeoVista Studio [11], SOM and Geovisualization examples in health [12], GEO-SOM [13, 14], and SAM-SOM* and MAM-SOM for Similarity Information Retrieval [15].

 The need to provide fast convergence as we exploit massive digital datasets led to the formulation of an updating rule for SOM. The mathematically improved learning-self organizing map (MIL-SOM) algorithm offers significant improvements both in terms of computational efficiency and quantization error. Standard SOM is a very popular visualization and clustering algorithm and is already well established so our primary focus is to explore proportional-integral-derivative control theory for speeding-up.

As frequently cited in the neural networks literature, SOM is a special architecture of neural networks that cluster the high-dimensional data vectors according to a similarity measure [4]. SOM clusters the data in a manner similar to cluster analysis but has an additional benefit of ordering the clusters and enabling the visualization of large numbers of clusters [16, 17]. This technique is particularly useful for the analysis of large datasets where similarity matching plays a very important role [4, 6]. SOM is used to classify the objects based on a measure of their similarities into groups thereby discovering the structure of the data hidden in large datasets [17–19]. It compresses information while preserving the topological and metric relationships of the primary data items [18]. The selection of the size of the map and the parameters used in estimation are key primary concerns in SOM training [17, 18].

 Although a few SOM studies have suggested improvements or undertaken a couple of enhancements, there is still little information available regarding the speed and quality of clusters, output choice of the number of output neurons, and updating procedure for output neurons. Earlier efforts by Lampinen and Oja [20] introduced a probing algorithm to solve complex distance calculations while yielding the best matching unit. Haese and vom Stein [21] proposed a better training regime using spline interpolation of the lattice of the map to reduce time complexity. In 2000, Su and Chang [22] suggested a three-stage method of incorporating *k*-means with SOM to reduce the cumbersome search process. The efforts of Kinouchi and Takada [23] yielded a quick learning idea for batch-learning SOM so that the learning process did not have to depend on the input order. Conan-Guez et al. [24] published a paper on a fast algorithm and implementation of dissimilarity of SOM culminating into a significant reduction in computational cost. Wu and Takatsuka [25] proposed an interesting solution to the border effect in SOM. Recent trends point to significant developments in terms of time complexity of SOM algorithm; however, this study introduces another SOM variant based on proportional-integral-derivative (PID) control theory, whose computational performance is fast and has low quantization error. The pressing demand for computationally rich and data-rich algorithms and renewed emerging interest in applications dealing with locational information are key motivating factors for undertaking this study.

The PID control with its three-term functionality offers a very attractive generic and computationally efficient solution to real world control problems [26–29] so there is a need to explore it in SOM context. PID control is the most widely used control strategy today [30] and provides simplicity, clear functionality, and ease of use [31]. Its core functionality includes (1) the proportional correcting term gives an overall control action relative to the measured error value; (2) the integral correcting term yields zero steady-state error in tracking a constant setpoint, a result frequently explained in terms of the internal model principle and demonstrated using the final value theorem; (3) the derivative correcting term recovers transient response through high-frequency compensation [30, 31].

The application of PID control to SOM can help with the visual exploration of disease and healthcare informatics datasets. Undertaking rapid, robust, and relevant analysis using an enhanced algorithm in supporting the decision-making process, particularly in domains that require timely, geospatial information [32–35], provides a solid basis for instantaneous access to modified value-added data and knowledge. This is further compounded by massive digital datasets that are being generated by tracking and reporting systems, improved geotechnologies, web-based portal systems, interoperable systems, and real-time data-rich environments. Although recent developments offer new opportunities to the research community, little attention has been paid, specifically, to algorithm development for visualizing and analyzing explanatory factors that explain health outcomes relative to the environment. For instance, the integration of algorithmic-trained data with GIS data models—particularly for physical database design efforts [36, 37], Similarity Information Retrieval [15], and building and exploring homogenous spatial data [13, 14]—may offer enormous benefits for the design, implementation, and extended use of SOM algorithms.

The basic idea for undertaking this study is motivated by an increased need to develop algorithms that can converge faster (an approach towards a definite value) and more efficiently than conventional techniques. The MIL-SOM algorithm, which possesses these key properties, is tested on four training geographic datasets representing biomedical and disease informatics application domains.

 The remainder of this paper is organized as follows. In Section 2, algorithm development is presented followed by subsections covering the primary structure of MIL-SOM algorithm and supplementary improvements. In Section 3, the data and methods for this study are presented.  Section 4 follows with the presentation of results and discussions. Lastly, concluding remarks and future implications are provided in Section 5.

## 2. Algorithm Development

### 2.1. The Basic Structure of MIL-SOM Algorithm

**Figure 1:**
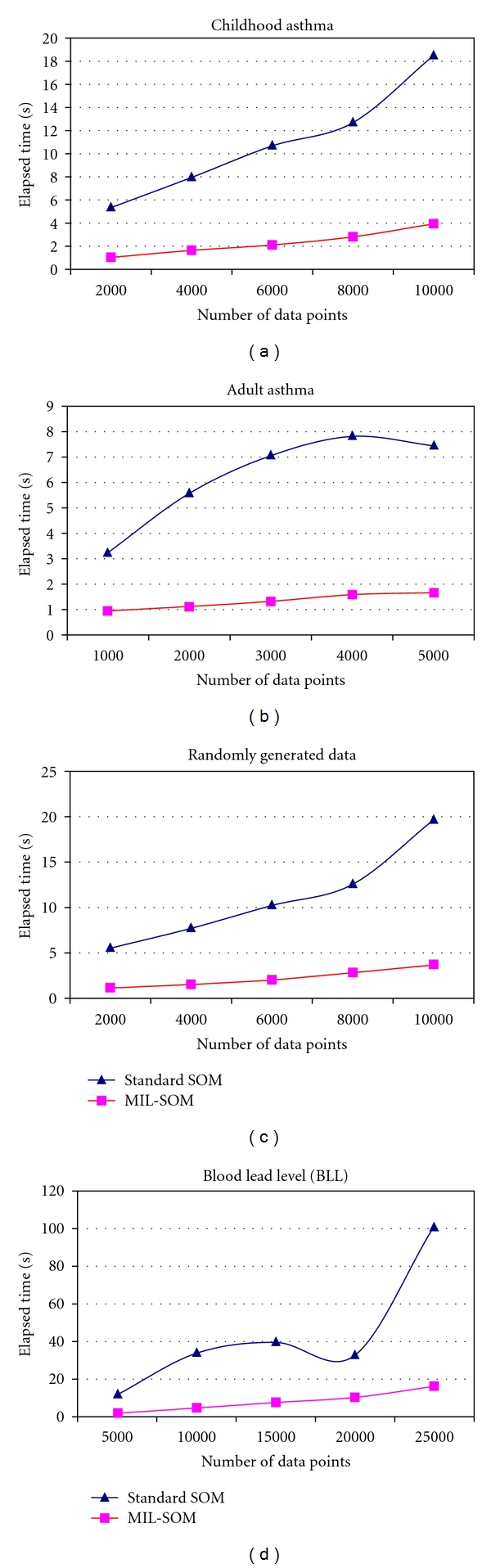
A comparison of Standard SOM and MIL-SOM algorithms using runtime versus the number of data points. The newly developed MIL-SOM algorithm converges faster than the standard SOM in all of the four training datasets used in the experiment.

The significant feature of this algorithm is the change in the weights of Kohonen's SOM through using the full blown PID control law thus offering more response control and faster convergence. This system employs a PID control to obtain optimal parameters for the MIL-SOM algorithm and to achieve the fast minimization of the difference between the desired output and the measured value. The main benefits of this algorithm include minimal additional computations per learning step, which are conveniently easy to implement. In terms of computational complexity, its learning/training process is similar to Kohonen's SOM. However, only a small fraction of the MIL-SOM algorithm has to be modified during each training step, adaptation is fast and the elapsed time is low, even if a large number of iterations might be necessary or the dataset is unusually large. The MIL-SOM algorithm enjoys other properties: it is very stable and has increased performance and maximizes time available for processing data thus it is scalable and has independence of input and insertion sequence. The computational cost for SOM exhibits linear complexity (Figure 1) where *n* is the number of units on the map, which is normally much lower than the original data set size *X*. However, since the complexity of SOM training is in *O*(*n*), it is clear that for a given dataset size *X*, the relative computational cost for the MIL-SOM algorithm drastically improved and cuts the learning rate almost by five times. Even with the increase of map size and data points, the learning rate remains stable.

The basic structure of the MIL-SOM algorithm consists of five key steps. However, steps one through three are identical to standard SOM.

(1) Begin with an input vector *X* = [*X*_*k*_ = 1,…, *X*_*k*_ = *n*] with *d* dimensions represented by an input layer *w*_*ij*_ containing a grid of units (*m* × *n*) with *ij* coordinates.
(2) Define MIL-SOM training parameters (size, training rate, map grid, and neighborhood size). Equally important is the main principle in selecting the size because it is contingent upon the number of clusters and pattern, or the MIL-SOM structure; however, defining an initial network may no longer be necessary as illustrated by the growing neural gas example [38].
(3) Compute and select the winning neuron, or Best Matching Unit (BMU), based on a distance measure as illustrated in (1) and (2), respectively,
  (1) $$\begin{array}{c} \left|\left|X_{k}-w_{\text{bmu}}\right|\right|=\underset{ij}{\arg\min}\left\{\left|\left|X_{k}-w_{ij}\right|\right|\right\}. \end{array}$$
  In Equation (1), ||·|| is the absolute distance, *w*_bmu_ is the winning neuron, and *w*_*ij*_ corresponds to the coordinates on the grid of units.
(4) Introduce a new updating rule based on PID control theory. The opportunity for improvement of the SOM model lies in the fact that the update rule employs the difference [*e*_*i*_(*t*) = *X*_*k*_(*t*) − *w*_*i*_(*t*)] between the input vector and the winning output neuron. In Kohonen's updating rule shown in (2), *e*_*i*_(*t*) is the equivalent of proportional only control law and is slow to converge, yet by adding derivative (damps oscillations) and integral (algorithm uses recent and less recent information in future actions) terms, convergence could be significantly increased and become more stable. By using this new updating rule in (3), weight vectors are adjusted faster than in original Kohonen's updating procedure, although theoretically the new model requires more computing time for each adjustment, the significant time savings can easily be obtained more directly in terms of significantly less adjustments:
  (2) $$\begin{array}{cc} & w_{i}\left(t+1\right)\\ & \;=w_{i}\left(t\right)+\alpha \left(t\right)h_{ci}\left(t\right)\left[X_{k}\left(t\right)-w_{i}\left(t\right)\right]\text{ for}\;i\in N_{c}\left(t\right),\\ & w_{i}\left(t+1\right)=w_{i}\left(t\right)\text{ for}\;i\notin N_{c}\left(t\right). \end{array}$$
  Kohonen's updating rule in (2) can be modified as follows:
  (3) $$\begin{array}{cc} w_{i}\left(t+1\right) & =w_{i}\left(t\right)+\alpha \left(t\right)h_{ci}\left(t\right)u_{i}\left(t\right)\;\;\text{for}\;i\in N_{c}\left(t\right),\\ w_{i}\left(t+1\right) & =w_{i}\left(t\right)\;\text{for}\;i\notin N_{c}\left(t\right),\\ u_{i}\left(t\right) & =e_{i}\left(t\right)+a_{1}\frac{de_{i}\left(t\right)}{dt}+a_{2}\overset{t+1}{\underset{t}{\int }}e_{i}\left(t\right)dt,\\ e_{i}\left(t\right) & =X_{k}\left(t\right)-w_{i}\left(t\right). \end{array}$$
  Equation (3) with its set of PID adjustments can further be rewritten as
  (4) $$\begin{array}{cc} w_{i}\left(t+1\right) & =w_{i}\left(t\right)+h_{ci}\left(t\right)[\alpha \left(t\right)e_{i}\left(t\right)+\alpha_{1}\left(t\right)\frac{de_{i}\left(t\right)}{dt}\\ & \;\;\;\;\;\;\;\;\;\;\;\;+\alpha_{2}\left(t\right)\overset{t+1}{\underset{t}{\int }}e_{i}\left(t\right)dt]\\ & \;\;\;\;\text{for}\;\;i\in N_{c}\left(t\right),\\ & w_{i}\left(t+1\right)=w_{i}\left(t\right)\;\;\text{for }i\notin N_{c}\left(t\right),\\ & e_{i}\left(t\right)=X_{k}\left(t\right)-w_{i}\left(t\right),\\ & \alpha_{1}\left(t\right)=\alpha \left(t\right)a_{1},\;\;\alpha_{2}\left(t\right)=\alpha \left(t\right)a_{2}. \end{array}$$
  In (4), there are potentially three new adjustable parameters, namely, the original learning rate *α*(*t*) and two additional ones, namely, *α*_1_(*t*) and *α*_2_(*t*). *w*_*i*_(*t*) is the output vector with its winning output neuron *i* while *N*_*c*_(*t*) and *h*_*ci*_(*t*) are the neighborhood and neighborhood kernel functions, respectively. Note that *a*_1_ and *a*_2_ are nonnegative parameters, which when set to 0 yield the original SOM update; and (*de*_*i*_  (*t*))/*dt* will tend to zero as learning improves. As long as the time window [*t*, *t* + 1] increases with time and is strictly enclosed in the time horizon [0, final time], the integral ∫_*t*_^*t*+1^ *e*_*i*_(*t*)*dt* will tend to zero (assuming of course *e*_*i*_(*t*) tends to zero). For the above reasons, as a first approximation [*α*(*t*), *α*_1_(*t*), *α*_2_(*t*)] can be fixed at the beginning of the MIL-SOM algorithm so one is reasonably assured that convergence would be fast. The first approximation [*α*(*t*), *α*_1_(*t*), *α*_2_(*t*)] is identified, in the pseudocode in Algorithm 1 as [alpha, alpha1, and alpha2]. To gain stability and divergence, the values of value of alpha, alpha1, alpha2, and radius are decreased until they reach zero.
(5) Repeat steps 3 and 4 until complete convergence is realized for the MIL-SOM network.

### 2.2. Supplementary Improvements in MIL-SOM Algorithm

In this subsection, we report on a measure undertaken to employ the *J*-metric to optimally select the best clusters during the MIL-SOM training. The measure to realize the best clustering approach is implemented using (5): 

Metric

(5) $$J=\underset{\text{som}}{\min}\overset{\text{data}}{\underset{k}{\sum }}\overset{\text{nodes}}{\underset{i}{\sum }}||X_{k,\text{som}}-w_{i,\text{som}}||=\underset{\text{som}}{\min\;}J_{\text{som}}.$$

For each complete SOM run, the program calculates the sum of the distances *J*_som_ between all possible pairs of neural nodes and data points and the best SOM is one with the smallest sum *J*_som_.

To optimally select the most appropriate number of output neurons (SOM units) thereafter report the best clusters, our strategy is to systematically choose for a given SOM the number of output neuron nodes and terminate when the slope of the *J*_metric_ nears zero. At this stage, further addition of output neurons leads to a marginal reduction in the *J*_metric_ (5). The program constrains the number of neurons between lower and upper bounds, node^low^ and nodes^high^, and then solves the optimization problem using (6). There is a slight problem with this in part because the number of neurons is an integer variable so the solution of the optimization problem below would require a soft computing approach that assumes the number of neurons is a continuous variable. Moreover, the rounding up or down of the optimal value of neurons in order to obtain an integer number is not well regarded in the optimization community, since the rounded value may no longer be optimal:

(6) $$\begin{array}{cc} F & =\underset{\text{nodes}}{\min}\frac{dJ}{d\text{nodes}}\\ \text{subject}\;\text{to}\; & J=\overset{\text{data}}{\underset{k}{\sum }}\overset{\text{nodes}}{\underset{i}{\sum }}||X_{k}-w_{i}||\\ & \begin{array}{c} \text{node}{\text{s}}^{\text{low}}\leq \text{nodes}\leq \text{node}{\text{s}}^{\text{high}}. \end{array} \end{array}$$

While there are numerous deterministic global optimization algorithms, it would be computationally inefficient to proceed in this direction partly because of the measurement error in our datasets, PID control sensitivity, nonsmoothness of the functions, and the potentially large number of SOM units. It would be, therefore, desirable to employ a soft computing approach such as simulated annealing [39] to solve the optimization problem to near global optimum mainly because nodes are a discrete variable. Moreover, we could simply modify the simulated annealing update step to enforce a discrete update of the optimization variable. The simulated annealing update procedure was adjusted to facilitate the selection of an optimal number of SOM units. For each dataset, we run 50 iterations and selected the optimal number based on the smallest sum of *J*_som_. 

## 3. Methods and Materials

Three published disease datasets encoded with a vector data structure and a fourth dataset (random computer-generated dataset) were used to test the standard SOM and MIL-SOM algorithms (Table 1). The first and second datasets are physician-diagnosed cases of childhood and adult asthma, both possessing six dimensions. The third dataset (consisting of blood lead levels (BLL) for children living in the City of Chicago) is from the Lead Poisoning Testing and Prevention Program of the Chicago Department of Public Health (CDPH). This very large dataset containing in excess of 881,385 records includes all reported blood lead screenings for every individual tested from January 1, 1997 through December 31, 2003. Of these, forty-seven percent of subjects had been tested multiple times. The deduplication process reduced this dataset to 469,297 records, which were aggregated at two levels: census block (*n* = 24, 691) and census block groups (*n* = 2, 506). The dataset had more than 13 dimensions, and the fourth dataset, randomly generated using the computer, had seven dimensions.

The coding of the MIL-SOM clustering algorithm was accomplished using two computational tools: MATLAB 7.5 (The MathWorks, Inc., Natick, Massachusetts) and SOM Toolbox 2.0 for Matlab (SOM Project, Hut, Finland).

We conducted multiple experiments to explore and analyze the performance and efficiency of standard SOM and MIL-SOM algorithm together with GIS (geographic information systems) techniques using a large-scale high-dimensional clinically acquired geographic data. We built a topological structure representing the original surface by encoding the disease map by means of a 3D spherical mesh output. The neurons were positioned based on their weight vectors. The neuron which was the nearest one to the sampling point in a Euclidean distance measure was elected as a winning neuron. We ran several experiments using three disease datasets encoded with a vector data structure (point and polygon data structure) and a randomly generated dataset. In addition to encoded disease data, each map also contained unorganized sample points. In setting up the experiments, we first randomly selected either 1000 or 2000 data points from the whole dataset, then continued adding on the same number of data points (e.g., 1000, 2000, 3000, etc. or 2000, 4000, 6000, etc.) until the completion of training. We implemented different data ranges for the distinct datasets due to their different sizes and trained the three datasets using two algorithms.

To test the MIL-SOM prototype, we deliberately confined our initial experiments to three well-understood datasets in terms of dimensions (variables) and size (number of records) so as to effectively study its properties. The fourth dataset, however, was introduced in the experiment to further examine any other effects of applying the newly designed MIL-SOM algorithm. Close attention was paid to the configuration of key SOM parameters, which are total training length, scalability, map and neighborhood size, and other training parameters during the comparison of the standard SOM and MIL-SOM algorithms. Other experimental procedures and training parameters have been reported in an earlier report [40] and therefore will not be repeated here.

The first set of experiments was done using two published datasets [41, 42] containing geographically referenced individual data points of children (*n* = 10, 335) and adult patients (*n* = 4, 910) diagnosed with asthma. The second set of experiments was conducted using the randomly generated dataset (*n* = 10, 000), while the final set of experiments was based on another published BLL dataset. The BLL dataset was attractive to use because it was big in size and had multiple dimensions.

Several experiments were conducted of the available data and training files were constructed using a number of samples ranging from 75% to 1%. For these experiments, the learning rate ranged from 0.5 in the rough-tuning phase to 0.05 in the fine-tuning phase. The initial neighborhood radius was varied depending on the map size, but it is normally equivalent to half of the map size and was gradually reduced during the training phase until it reached 1. At any instant during the training, the minimum value of the neighborhood radius was 1, and 50 iterations were run to identify the best SOM cluster based on the smallest sum *J*_som_. *K*-means clustering method was used to partition and further investigate clusters. The SOM toolbox has a validation tool that integrates the *k*-means based on the Davies-Bouldin index. The clusters were post-processed using ESRI's ArcGIS software and final maps representing MIL-SOM clusters were created.

**Figure 2:**
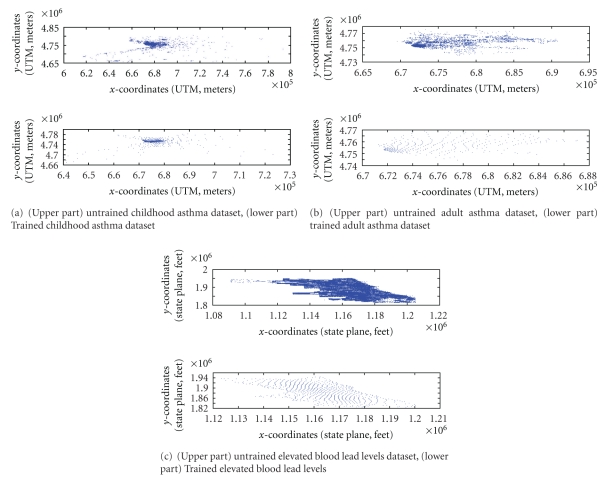
Figure 2(a)–2(c) illustrate the spatial distributions of untrained and MIL-SOM trained datasets.  Figure 1(a) represents adult asthma (map units in UTM, meters); Figure 2(b) is childhood asthma (map units in UTM, meters); Figure 2(c) is elevated blood lead levels (map units in state plane, feet).

At the end of each training session, it was vital to determine whether both the standard SOM and MIL-SOM matched with the trained data. Several ways to achieve this goal exist in the literature [16, 43], but we preferred to assess the quality of data representation by means of a *U*-Matrix and through a comprehensive analysis of map quality using two types of error: quantization error and topological error. They provided a sound basis to measure map quality [4, 5] of the four training datasets. In fact, quantization error facilitated the training process and returned a granularity level of data representation (mapping precision) for the training datasets, while the topological error evaluated how adjacent neurons were close to the first- and second-best-matching units or measured the proportionality of all data vectors in relation to first- and second-best-matching units. Simply, topological error considers the ratio of data vectors (neurons) for which the first- and second-best-matching units are not adjacent. The analysis of the neighborhood of the best matching unit is very informative because it provides insights regarding the occurrence of effective data representation; and knowledge of this fact elucidated whether the input data vectors had adapted well to the trained dataset. Figure 2(a)–2(c) illustrate the spatial distributions of untrained and MIL-SOM-trained datasets. From these figures, one can surmise that MIL-SOM-trained datasets effectively capture and accurately represent the original features of untrained datasets. 

Exploration of potential patterns in the trained datasets was further achieved through a comprehensive analysis of the *U*-Matrix [16, 43]. In general, the *U*-Matrix employs the distance matrices to visually represent the distances between neighboring network units (neurons) as a regular 2-dimensional grid of neurons. The *U*-Matrix shows the distances from each neuron's center to all of its neighbors. Typically, in the *U*-Matrix dark colorings between the neurons correspond to large distance in the input space, while the light coloring between neurons specifies that the vectors are close to each other. Component planes of both standard SOM and MIL-SOM algorithms were visualized further by slicing them to show each component, which aided on-screen-based probing and visual interpretations.

## 4. Results and Discussions

The significance of incorporating MIL-SOM clustering data into GIS provides an opportunity for better interpretation of combined geographic and medical data, which could lead to better formulation of study hypotheses. This study has been successful in implementing a mathematical improvement to resolve efficiency and convergence concerns associated with standard SOM. The algorithm works well and provides better knowledge exploration space than other techniques because it maintains the internal relationships between the data points, which are lost to a certain extent with other clustering algorithms when the results are mapped onto a lower dimensional plane.

**Figure 3:**
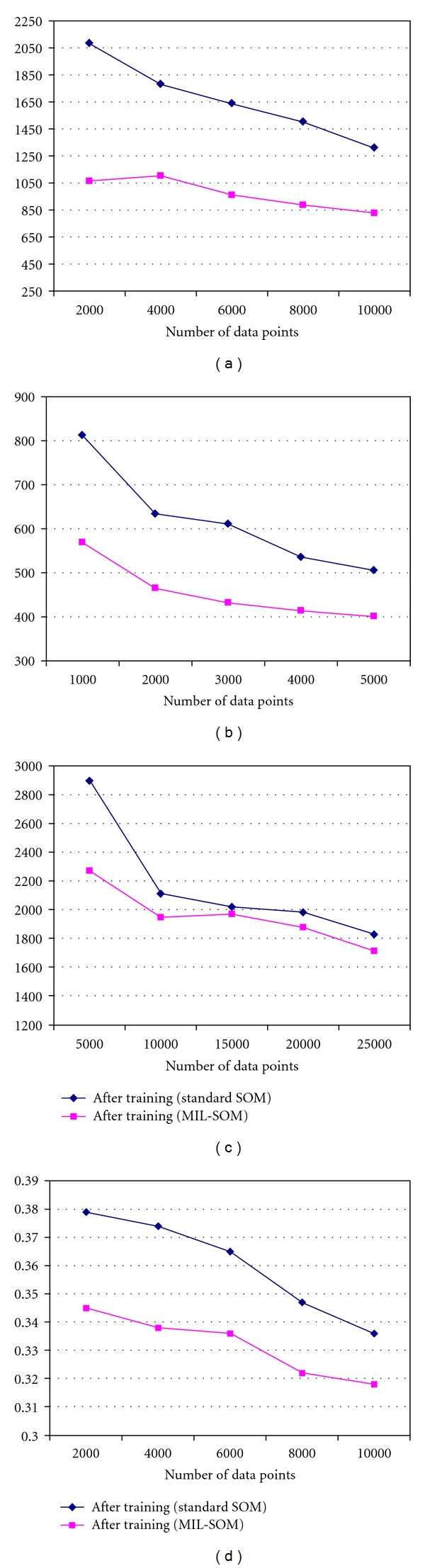
Figure 3(a)–3(d) illustrate experimental results for the standard SOM and MIL-SOM algorithms by comparing the number of data points and quantization error.

Figure 3(a)–3(d) illustrate experimental results for the standard SOM and MIL-SOM algorithms by comparing the number of data points and quantization error [40]. The data indicates a much more competitive MIL-SOM than standard SOM with respect to an overall decrease in quantization error. The quantization errors decreased in all cases but topological errors increased in 14 out of the 16 training regimes. From Table 2, one can surmise that the topological errors were very low for both algorithms, indicating that a sound map structure and a proper map folding was achieved for the trained datasets, which closely represented the input data vectors. Given that topological errors increased more than 100% for the randomly generated and BLL datasets, it is possible that standard SOM outperformed the MIL-SOM algorithm in terms of preserving topology. The highest topological error is recorded in the BLL datasets, followed by adult asthma dataset, then the randomly generated dataset; the least error is observed in childhood asthma dataset indicating that the neighbors, are closer. This may be a result of different map sizes and shapes. In our training, we utilized the rectangular grid and if the units were not neighbors then the topological error increased thus indicating the amount of noise in some of our datasets. It could also be because PID is sensitive to missing and noisy data [29], which may require further adjustments.

**Figure 4:**
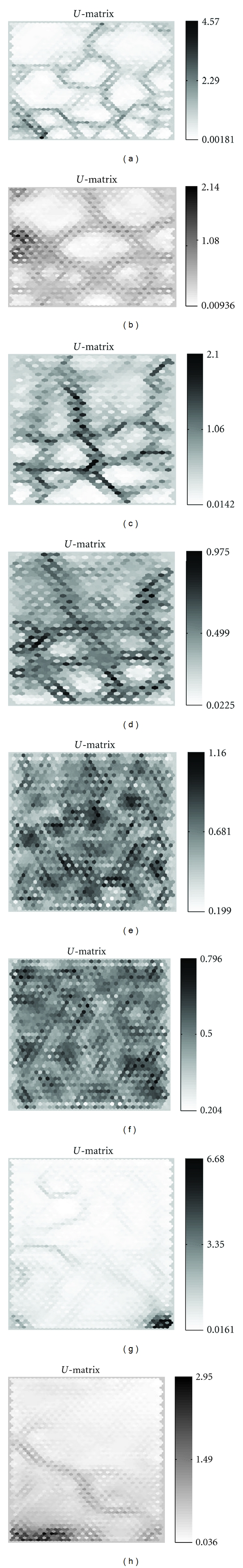
illustrates *U*-Matrices and clusters derived from the standard SOM ((a), (c), (e), and (g)) and MIL-SOM ((b), (d), (f), and (h)) algorithms. Experimental datasets include childhood asthma ((a), (b)); adult asthma ((c), (d)); computer random generated ((e), (f)); and blood lead levels ((g), (h)). Map size 40 × 40 neurons and the topology of the neurons are hexagonally in shape.

Figure 4 illustrates *U*-Matrices and clusters derived from the standard SOM and MIL-SOM algorithms. Onscreen display and probing of the *U*-Matrices revealed unique features, and it was quite evident that clusters presented using the MIL-SOM algorithm were more clearly separated than those of the standard SOM though in some cases (Figures 4(e) and 4(f)) this was not. The MIL-SOM algorithm also had a better depiction of the weight vectors of the neuron (as shown by a clear tone in light and dark coloring); simpler lower-dimensional spaces; a better projection, but a well-preserved input topology is still evident within the standard SOM than in MIL-SOM. Additional analyses and experimentations of cluster quality and size of the trained datasets are required to better understand other unique features of the MIL-SOM algorithm.

**Figure 5:**
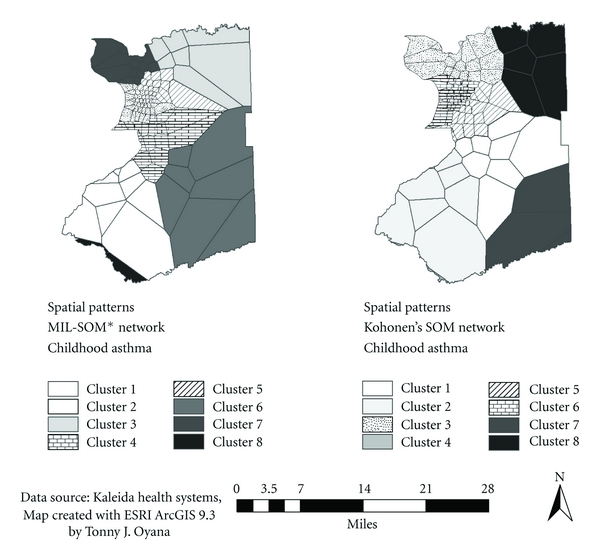
Cluster distributions showing delineated regions of childhood asthma using the MIL-SOM (major clusters 2, 4, and 5) and Kohonen's SOM algorithms (major clusters 3, 5, and 6).

**Figure 6:**
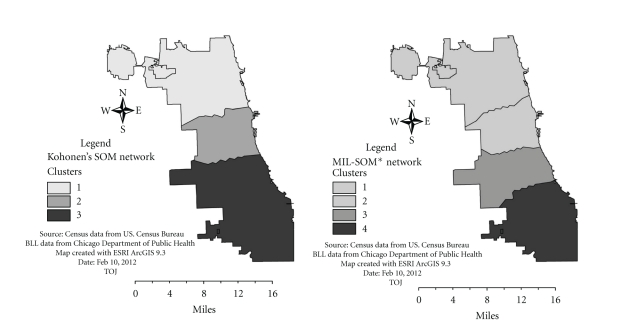
Cluster distributions showing delineated regions of elevated blood lead levels using the MIL-SOM (major clusters are 2, 3, and 4) and Kohonen's SOM algorithms (major clusters 2 and 3).

Figures 5 and 6 present maps of spatial patterns and clusters derived from Kohonen's SOM and MIL-SOM algorithms. The maps provide very interesting spatial patterns and unique features for the MIL-SOM algorithms.

- For Figure 5, the major clusters (these were identified during post-processing) are 2, 4, and 5; and 3, 5, and 6 for MIL-SOM and Kohonen's SOM algorithms, respectively. Although clusters of childhood asthma are similar to adult asthma, there is a wide spatial distribution of these clusters in the Westside, Downtown, and Eastside signifying the severity of asthma among children. This finding is consistent with the previous ones [42, 44]. There are notable spatial differences between the geographic extent of the clusters generated by MIL-SOM and Kohonen's SOM algorithms because they are a good fit for epidemiological studies.
- The major clusters for adult asthma are 2, 6, and 7; and 3, 4, and 7 using MIL-SOM and Kohonen's SOM algorithms, respectively. The major clusters of adult asthma are located in Downtown, Westside, and to a less extent in the Eastside of the City of Buffalo, New York. These clusters are consistent with previous findings [41, 44, 45] that applied traditional epidemiological methods to investigate the prevalence of adult asthma. Overall, the identified three subsets (geographic regions) of adult asthma are similar to the ones identified in childhood asthma, MIL-SOM algorithms provide tighter clusters than Kohonen's SOM.
- For Figure 6, the major clusters are 2, 3, and 4; and 2 and 3 for MIL-SOM and Kohonen's SOM, respectively, occurring in the Westside, Eastside, and Downtown areas of the City of Chicago, Illinois. Cluster 1 in both maps is a minor one representing very low blood lead levels in the North of the City of Chicago. These homogenous regions are consistent with the findings from a previous study that applied traditional epidemiological methods to investigate the prevalence of elevated blood levels. Clusters derived from both algorithms are strikingly similar, with the exception of the fourth cluster from the MIL-SOM algorithm. The MIL-SOM algorithm has identified three subsets (geographies) of elevated blood levels and one reference geography (area showing low levels), which require additional evaluation.

While the main properties of the MIL-SOM clustering algorithm have been reported earlier, it is equally important to reinforce further that this algorithm is fast and computationally efficient. Key findings based on this prototype show successful performance in terms of computational speed and high map quality output. This algorithm is useful for knowledge discovery and the classification of large-scale geographical datasets.

## 5. Conclusions and Future Work

The new heuristics MIL-SOM algorithm was derived from Kohonen's SOM. It provides a better updating procedure than Kohonen's SOM. In particularly, this clustering algorithm resolves four key issues: (1) enhancing the speed and quality of clustering, (2) selecting the best clusters using the *J*_metric_, (3) the updating procedure for the winning neurons, and (4) increasing the learning rate in the SOM model. This algorithm has great potential to analyze large-scale geographical datasets and any other dataset and can be used to visually identify and categorize such datasets into similar groups that share common properties.

The findings show that the MIL-SOM algorithm is a multifaceted technique in that it can be used both as a visualization and clustering tool. The algorithm offers improvements in terms of computational efficiency and low quantization error. Other key properties include the fact that it is computationally fast, robust, and it returns a high map quality output. Its core competitiveness (or competence) includes it being a faster convergence tool for the visual exploration of multivariate data, which allows for a rapid cluster exploration thus enabling a reduced computational cost; and it is affluent regarding weight vector initialization and preserves original attribute space.

Although the PID control approach has offered a key benefit of fast convergence for the MIL-SOM algorithm, there are some limitations associated with its controls. For example, the value that corresponds to the desired output in the proposed learning rule is currently under investigation. Other future plans include the need to measure statistical significance and further validation of the MIL-SOM algorithm. Current work is primarily focused on:

- extending MIL-SOM to very large datasets with many dimensions;
- exploring process gains in PID control and separately comparing the PID control with MIL-SOM approach using problematic/noisy datasets;
- exploring MIL-SOM algorithm together with a new delineation *FES*-*k*-means algorithm [46, 47].

The MIL-SOM algorithm has broad implications for knowledge discovery and visualization for disease and health informatics because of its flexibility and its ability to identify complex structures that do not fit predefined hypotheses. It has the potential for increasing the quality of health outcomes relative to the environment. The algorithm serves as catalyst to develop fully integrated analytical environments with functionalities to enable advanced spatial analysis, spatial data mining, summarization capabilities, and visual analytics. Its design and implementation in a GIS setting may very well serve numerous purposes such as facilitating Similarity Information Retrieval and the identification of homogenous units. It may support the exploration of publicly available large scale health databases. The Centers for Disease Control and Prevention (CDC) and many of these federal agencies have standardized the collection of disease and health data and as a result they have established large and ontologically coherent surveillance databases that now incorporate location information.

## Figures and Tables

**Algorithm 1:**
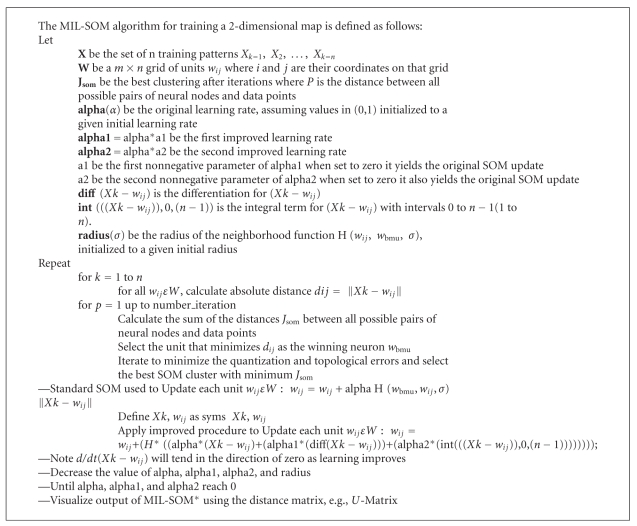
Presents the pseudocode for the MIL-SOM (Mathematically Improved Learning) Algorithm.

**Table 1:** Description of experimental datasets.

Datasets	Number of input dimensions	Number of records	Description of input dimensions
Childhood asthma	6	10335	X and Y coordinates;
			case control value;
			residence distance (500 m)
			of a patient to a highway,
			to a pollution source (1000 m),
			to a sampling site of measured particulate
			matter concentrations (1000 m).
Adult asthma	6	4910	Unknown organized spatial
			patterns and is noisy
Randomly generated	7	10000	Age of housing units is given in percentile
			intervals beginning with pre-1939
			units up to the year 2000 (9 dimensions);
			median year; elevated blood lead levels for
Elevated blood lead levels	15	2506, 24691	1997, 2000, and 2003; X and Y coordinates

**Table 2:** Training parameters of standard SOM and MIL-SOM algorithms for experimental datasets.

Data points	Standard SOM	MIL-SOM	Standard SOM		MIL-SOM	
	Elapsed time (s)	Elapsed time (s)	Qe	Te	Qe	Te
Childhood asthma data*						
2000	5.406	1.016	2081	0.052	1064	0.019
4000	8.016	1.625	1780	0.032	1103	0.035
6000	10.75	2.094	1637	0.028	961	0.036
8000	12.75	2.797	1502	0.029	887	0.031
10000	18.547	3.922	1309	0.031	828	0.037

Adult asthma data*						
1000	3.25	0.922	813	0.034	571	0.021
2000	5.61	1.094	635	0.007	467	0.039
3000	7.109	1.297	612	0.018	434	0.04
4000	7.875	1.563	537	0.015	416	0.031
4910	7.5	1.641	507	0.019	403	0.043

Randomly generated data*						
2000	5.625	1.141	0.379	0.065	0.345	0.107
4000	7.813	1.516	0.374	0.059	0.338	0.127
6000	10.344	2.016	0.365	0.057	0.336	0.147
8000	12.672	2.828	0.347	0.065	0.322	0.141
10000	19.765	3.703	0.336	0.062	0.318	0.136

Blood lead levels data**						
5000	12.435	2.018	2897.2	0.0032	2271.2	0.0094
10000	34.435	4.808	2110.7	0.0047	1947	0.0185
15000	40.134	7.748	2018.1	0.0074	1969.5	0.022
20000	33.193	10.418	1980.5	0.0057	1877.4	0.0214
24691	101.035	16.276	1826.8	0.0083	1712.9	0.0211

*Note. There was great improvement in map quality in terms of the quantization errors (Qe) for MIL-SOM for real-world datasets, but standard SOM appears to do better with the topological errors (Te), especially for the random dataset that is noisy. The initial neighborhood radii for the rough training phase and fine-tuning phase were set as max(m size)/4 = 5 and max(m size)/4)/4 = 1.25, respectively, until the fine-tuning radius reached 1, where max is the maximum value of the map size matrix. For all the datasets, the map size was (20 20), so max was 20. Some minor adjustments were initially made during the MIL-SOM training with respect to the specifications of map size, neighborhood radius, and the length of training to fine tune the training SOM parameters [40].

**Training parameters for the new training dataset—blood lead levels (BLLs). The results further confirm the trends reported in an earlier report [40].
